# Instruments for Assessing Risk of Bias and Other Methodological Criteria of Published Animal Studies: A Systematic Review

**DOI:** 10.1289/ehp.1206389

**Published:** 2013-06-14

**Authors:** David Krauth, Tracey J. Woodruff, Lisa Bero

**Affiliations:** 1 Department of Clinical Pharmacy, and; 2 Department of Obstetrics, Gynecology, and Reproductive Sciences, University of California, San Francisco, San Francisco, California, USA; 3 Program on Reproductive Health and the Environment, Oakland, California, USA; 4 Institute for Health Policy Studies, University of California, San Francisco, San Francisco, California, USA

## Abstract

Background: Results from animal toxicology studies are critical to evaluating the potential harm from exposure to environmental chemicals or the safety of drugs prior to human testing. However, there is significant debate about how to evaluate the methodology and potential biases of the animal studies. There is no agreed-upon approach, and a systematic evaluation of current best practices is lacking.

Objective: We performed a systematic review to identify and evaluate instruments for assessing the risk of bias and/or other methodological criteria of animal studies.

Method: We searched Medline (January 1966–November 2011) to identify all relevant articles. We extracted data on risk of bias criteria (e.g., randomization, blinding, allocation concealment) and other study design features included in each assessment instrument.

Discussion: Thirty distinct instruments were identified, with the total number of assessed risk of bias, methodological, and/or reporting criteria ranging from 2 to 25. The most common criteria assessed were randomization (25/30, 83%), investigator blinding (23/30, 77%), and sample size calculation (18/30, 60%). In general, authors failed to empirically justify why these or other criteria were included. Nearly all (28/30, 93%) of the instruments have not been rigorously tested for validity or reliability.

Conclusion: Our review highlights a number of risk of bias assessment criteria that have been empirically tested for animal research, including randomization, concealment of allocation, blinding, and accounting for all animals. In addition, there is a need for empirically testing additional methodological criteria and assessing the validity and reliability of a standard risk of bias assessment instrument.

Citation: Krauth D, Woodruff TJ, Bero L. 2013. Instruments for assessing risk of bias and other methodological criteria of published animal studies: a systematic review. Environ Health Perspect 121:985–992 (2013); http://dx.doi.org/10.1289/ehp.1206389

## Introduction

Results from animal toxicology studies are a critical—and often the only—input to evaluating potential harm from exposure to environmental chemicals or the safety of drugs before they proceed to human testing. However, there is significant debate about how to use animal studies in risk assessments and other regulatory decisions (Adami et al. 2011; European Centre for Ecotoxicology and Toxicology of Chemicals 2009; Weed 2005; Woodruff and Sutton 2011). An important part of this debate is how to evaluate the methodology and potential biases of the animal studies in order to establish how confident one can be in the data.

For the evaluation of human clinical research, there is a distinction between assessing risk of bias and methodological quality (Higgins and Green 2008). Risks of bias are methodological criteria of a study that can introduce a systematic error in the magnitude or direction of the results (Higgins and Green 2008). In controlled human clinical trials testing the efficacy of drugs, studies with a high risk of bias—such as those lacking randomization, allocation concealment, or blinding of participants, personnel, and outcome assessors—produce larger treatment effect sizes, thus falsely inflating the efficacy of the drugs compared with studies that have these design features (Schulz et al. 1995; Schulz and Grimes 2002a, 2002b). Biased human studies assessing the harms of drugs are less likely to report statistically significant adverse effects (Nieto et al. 2007). An assessment of a study’s methodology includes evaluation of additional study criteria related to how a study is conducted (e.g., in compliance with human subjects guidelines) or reported (e.g., study population described). Finally, risk of bias is not the same as imprecision (Higgins and Green 2008). Whereas bias refers to systematic error, imprecision refers to random error. Although smaller studies are less precise, they may not be more biased.

Although there is a well-developed and empirically based literature on how to evaluate the risk of bias of randomized controlled clinical trials, less is known about how to do this for animal studies. Some risks of bias in animal studies have been identified empirically. For example, analyses of animal studies examining interventions for stroke, multiple sclerosis, and emergency medicine have shown that lack of randomization, blinding, specification of inclusion/exclusion criteria, statistical power, and use of comorbid animals are associated with inflated effect estimates of pharmaceutical interventions (Bebarta et al. 2003; Crossley et al. 2008; Minnerup et al. 2010; Sena et al. 2010; Vesterinen et al. 2010). However, these studies used a variety of instruments to evaluate the methodology of animal studies and often mixed assessment of risks of bias, reporting, and other study criteria.

Several guidelines and instruments for evaluating the risks of bias and other methodological criteria of animal research have been published, but there has been no attempt to compare the criteria that they include; to determine whether risk of bias, reporting, or other criteria are assessed; or to determine whether the criteria are based on empirical evidence of bias. The purpose of this review was 2-fold: *a*) to systematically identify and summarize existing instruments for assessing risks of bias and other methodological criteria of animal studies, and *b*) to highlight the criteria that have been empirically tested for an association with bias in either animal or clinical models.

## Methods

*Inclusion/exclusion criteria*. Articles that met the following inclusion criteria were included: *a*) The article was a published report focusing on the development of an instrument for assessing the methodology of animal studies, and *b*) the article was in English. Where multiple analyses using a single instrument were published separately, the earliest publication was used. Modifications or updates of previously published instruments were considered new instruments and included. We did not include applications of previously reported instruments that were used, for example, to assess a certain area of animal research.

*Search strategy*. We searched Medline for articles published from January 1966 through November 2011 using a search term combination developed with input from expert librarians. Bibliographies from relevant articles were also screened to find any remaining articles that were not captured from the Medline search. Our search strategy contained the following MeSH terms, text words, and word variants:

{(animal experimentation[mh]) AND (standards[sh] OR research design[mh] OR bias[tw] OR biases[tw] OR checklist*[tw] OR translational research/ethics)} OR {(animals, laboratory[majr] OR disease models, animal[mh] OR drug evaluation, preclinical[mh] OR chemical evaluation OR chemical toxicity OR chemical safety) AND (research[majr:noexp] OR translational research[majr] OR research design[majr] OR “quality criteria”) AND (guideline* OR bias[tw] OR biases[tiab] OR reporting[tw])} OR {(animal*[ti] OR preclinical[ti] OR pre-clinical[ti] OR toxicology OR toxicological OR ecotoxicology OR environmental toxicology) AND (methodological quality OR research reporting OR study quality OR “risk of bias” OR “weight of evidence”)} OR {(CAMARADES[tiab] OR “gold standard publication checklist” OR exclusion inclusion criteria animals bias) OR (peer review, research/standards AND Animals[Mesh:noexp])} OR {(models, biological[mh] OR drug evaluation, preclinical[mh] OR toxicology[mh] OR disease models, animal[majr]) AND (research design[mh] OR reproducibility of results[mh] OR “experimental design”) AND (quality control[mh] OR guidelines as topic[mh] OR bias[tw] OR “critical appraisal”) AND (Animals[Mesh:noexp])} AND eng[la].

*Article selection*. Studies were screened in two stages. Initially, we reviewed abstracts and article titles, and only those articles meeting our inclusion criteria were further scrutinized by reading the full text. Any articles that did not clearly meet the criteria after review of the full text were discussed by two authors, who made the decision about inclusion. Exact article duplicates were removed using Endnote X2 software (Thomson Reuters, Carlsbad, CA).

*Data extraction*. We extracted data on each criterion included in each instrument, as well as information on how the instrument was developed.

*Instrument development and characteristics*. We recorded the method used to develop each instrument (i.e., whether the criteria in the instrument were selected based on consensus, previous animal instruments, and/or clinical instruments). We also recorded whether or not the criteria in the instrument were empirically tested to determine if they were associated with biased effect estimates. Empirical testing was rated as completed if at least one of the individual criterion was empirically tested.

Numerical methodological “quality” scores have been shown to be invalid for assessing risk of bias in clinical research (Jüni et al. 1999). The current standard in evaluating clinical research is to report each component of the assessment instrument separately and not calculate an overall numeric score (Higgins and Green 2008). Although the use of quality scores is now considered inappropriate, it is still a common practice. Therefore, we also assessed whether and how each instrument calculated a “quality” score.

We also noted whether the instrument had been tested for reliability and validity. Reliability in assessing risk of bias refers to the extent to which results are consistent between different coders or in trials or measurements that are repeated (Carmines and Zeller 1979). Validity refers to whether the instrument measures what it was intended to measure, that is, methodological features that could affect research outcomes (Golafshani 2003).

*Study design criteria to assess risk of bias and other methodological criteria*. Based on published risk of bias assessment instruments for clinical research, we developed an *a priori* list of criteria and included additional criteria if they occurred in the review of the animal instruments (Cho and Bero 1994; Higgins and Green 2008; Jadad et al. 1996; Schulz et al. 2010).

We collected risk of bias, methodological, and reporting criteria because these three types of assessment criteria were often mixed in the individual instruments. The final list of these criteria is as follows:

Treatment allocation/randomization. Describes whether or not treatment was randomly allocated to animal subjects so that each subject has an equal likelihood of receiving the intervention.
Concealment of allocation. Describes whether or not procedures were used to protect against selection bias by ensuring that the treatment to be allocated is not known by the investigator before the subject enters the study.
Blinding. Relates to whether or not the investigator involved with performing the experiment, collecting data, and/or assessing the outcome of the experiment was unaware of which subjects received the treatment and which did not.
Inclusion/exclusion criteria. Describes the process used for including or excluding subjects.
Sample size calculation. Describes how the total number of animals used in the study was determined.
Compliance with animal welfare requirements. Describes whether or not the research investigators complied with animal welfare regulations.
Financial conflict of interest. Describes if the investigator(s) disclosed whether or not he/she has a financial conflict of interest.
Statistical model explained. Describes whether the statistical methods used and the unit of analysis are stated and whether the statistical methods are appropriate to address the research question.
Use of animals with comorbidity. Describes whether or not the animals used in the study have one or more preexisting conditions that place them at greater risk of developing the health outcome of interest or responding differently to the intervention relative to animals without that condition.
Test animal descriptions. Describes the test animal characteristics including animal species, strain, substrain, genetic background, age, supplier, sex, and weight. At least one of these characteristics must be present for this criterion to be met.
Dose–response model. Describes whether or not an appropriate dose–response model was used given the research question and disease being modeled.
All animals accounted for. Describes whether or not the investigator accounts for attrition bias by providing details about when animals were removed from the study and for what reason they were removed.
Optimal time window investigated. Describes whether or not the investigator allowed sufficient time to pass before assessing the outcome. The optimal time window used in animal research should reflect the time needed to see the outcome and depends on the hypothesis being tested. The optimal time window investigated should not be confused with the “therapeutic time window of treatment,” which is defined as the time interval after exposure or onset of disease during which an intervention can still be effectively administered (Candelario-Jalil et al. 2005).

We extracted data on the study design criteria assessed by each instrument. We recorded the number of criteria assessed for each instrument, excluding criteria related only to journal reporting requirements (i.e., headers in an abstract).

*Analysis.* Here we report the frequency of each criterion assessed, as well as the frequency of any additional criteria that were included in the instruments.

## Results

**Figure 1:**
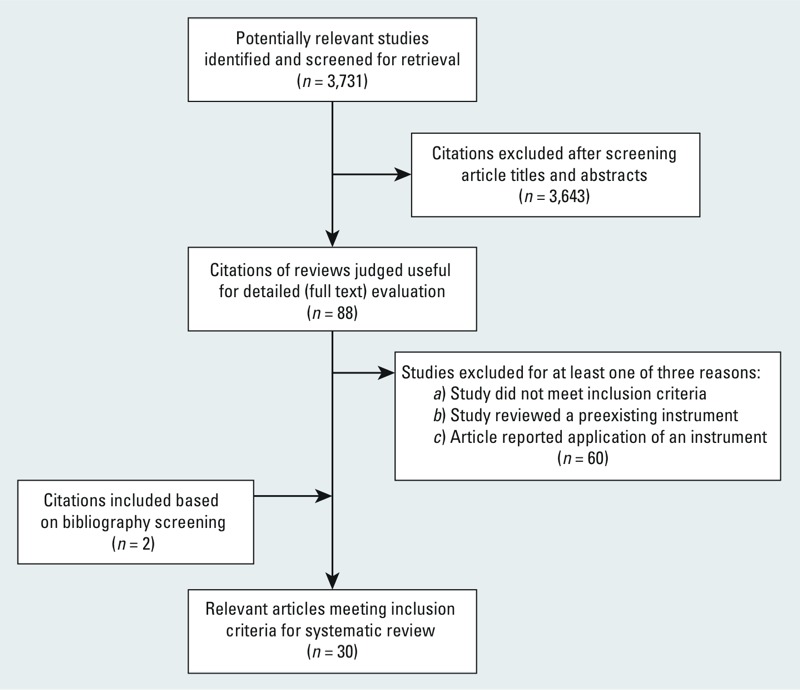
Flow of included studies. *n* indicates the number of studies.

As shown in Figure 1, we identified 3,731 potentially relevant articles. After screening the article titles and abstracts, we identified 88 citations for full text evaluation. After reviewing full text, 60 papers were excluded for at least one of three reasons: *a*) They did not meet inclusion criteria; *b*) the studies reviewed a preexisting instrument; and *c*) the article reported application of an instrument. After screening bibliographies, two additional instruments were found. Overall, 30 instruments were identified and included in the final analysis.

Table 1 lists the criteria of each instrument. Of the 30 instruments, 13 were derived by modifying or updating previously developed animal research methodology assessment instruments or citing animal studies supporting the inclusion of specific criteria; 3 were derived from previously developed clinically based risk of bias assessment instruments or citing clinical studies supporting the inclusion of specific criteria; 5 were developed using evidence from clinical research and either through consensus or citing past instrument publications; 3 were developed through consensus and citing past publications; and 6 had no description of how they were developed.

**Table 1:** Description of instruments for assessing risk of bias and methodological criteria of animal studies (*n* = 30).

Instrument identifier	Method used to develop instrument	No. of criteria	Quality score calculated	Specific disease modeled	Instrument criteria empirically tested	Intended use of instrument
Vesterinen etal. 2011	Developed using evidence from clinical research and either through consensus or citing past animal instrument publications. Instrument development was based on previous research studies and new criteria not captured by past publications.	12	No	None	No	Preclinical drug research
Agerstrand etal. 2011	Based on consensus and citing past guidelines. Authors collaborated with researchers and regulators to develop the criteria, relied on previously published reports, drew from their own professional experiences, and received additional suggestions from ecotoxicologists from Brixham Environmental Laboratories/AstraZeneca and researchers within the MistraPharma research program.	25	No	None	No	Environmental toxicology research (specifically environmental risk assessment of pharmaceuticals)
National Research Council Institute for Laboratory Animal Research 2011	Derived by modifying or updating previously developed animal research methodology assessment instruments or citing animal studies supporting the inclusion of specific criteria. Evidence-based rationale for including specific criteria is provided. Expert laboratory animal researchers with scientific publishing experience formed the committee that developed these guidelines.	19	No	None	No	General animal research
Lamontagne etal. 2010	Developed using evidence from clinical research and either through consensus or citing past animal instrument publications; relied on the PRISMA (Preferred Reporting Items for Systematic Reviews and Meta-Analyses) Statement for determining relevant risk of bias criteria. Some of the criteria were incorporated into the risk of bias assessment based on clinical evidence showing an association between the criterion and overestimated treatment effect (Montori etal. 2005).	9	No	Sepsis	No	Preclinical drug research
Conrad and Becker 2010	Developed through consensus and citing past guidelines; constructed using five previously developed quality assessment guidelines.	10	Yes^*a*^	None	No	General animal research
Vesterinen etal. 2010	Derived by modifying or updating previously developed animal research methodology assessment instruments or citing animal studies supporting the inclusion of specific criteria; derived from the consensus statement “Good Laboratory Practice” for modeling stroke (Macleod etal. 2009).	5	No	Multiple sclerosis	Yes	Preclinical drug research
Kilkenny etal. 2010 (the ARRIVE Guidelines)	Developed using evidence from clinical research and either through consensus or citing past animal instrument publications; developed using the CONSORT (CONsolidated Standards of Reporting Trials) criteria, consensus, and consultation among scientists, statisticians, journal editors, and research funders.	13	No	None	No	General animal research
Minnerup etal. 2010	Derived by modifying or updating previously developed animal research methodology assessment instruments or citing animal studies supporting the inclusion of specific criteria; derived from the STAIR (Stroke Therapy Academic Industry Roundtable) recommendations (STAIR 1999).	11	Yes^*b*^	Stroke	No	Preclinical drug research
Hooijmans etal. 2010 (the gold standard publication checklist; GSPC)	Derived by modifying or updating previously developed animal research methodology assessment instruments or citing animal studies supporting the inclusion of specific criteria. Many of the criteria in the GSPC are supported by previous studies showing the importance of such parameters. The authors also discussed and optimized the GSPC with animal science experts.	17	No	None	No	General animal research
van der Worp etal. 2010	Developed using evidence from clinical research and either through consensus or citing past animal instrument publications; recommendations based largely on CONSORT and to a smaller extent on animal guidelines (Altman etal. 2001; Dirnagl 2006; Macleod etal. 2009; Sena etal. 2007; STAIR 1999).	9	No	Stroke	No	Preclinical drug research
Macleod etal. 2009	Developed using evidence from clinical research and either through consensus or citing past animal instrument publications; criteria based on past meta-analyses done by CAMARADES (Collaborative Approach to Meta-Analysis and Review of Animal Data from Experimental Studies) researchers and CONSORT.	9	No	Stroke	No	Preclinical drug research
Fisher etal. 2009	Derived by modifying or updating previously developed animal research methodology assessment instruments or citing animal studies supporting the inclusion of specific criteria; updated the original STAIR guidelines (STAIR 1999). No description of how the new instrument was developed.	15	No	Stroke	No	Preclinical drug research
Rice etal. 2008	Derived from previously developed clinically based risk of bias assessment instruments or citing clinical studies supporting the inclusion of specific criteria; modified form of the Jadad criteria (Jadad etal. 1996) used to assess clinical interventions.	6	No	Animal pain models	No	Preclinical drug research
Sniekers etal. 2008	No description of how the instrument was developed.	7	No	Osteoarthritis	Yes	Preclinical drug research
Sena etal. 2007	Derived by modifying or updating previously developed animal research methodology assessment instruments or citing animal studies supporting the inclusion of specific criteria; derived from four previous checklists: STAIR (1999), Amsterdam criteria (Horn etal. 2001), CAMARADES (Macleod etal. 2004), and Utrecht criteria (van der Worp etal. 2005).	21	No	Stroke	Yes	Preclinical drug research
Unger 2007	No description of how the instrument was developed.	4	No	None	No	Preclinical drug research
Hobbs etal. 2005	Derived by modifying or updating previously developed animal research methodology assessment instruments or citing animal studies supporting the inclusion of specific criteria; modified version of Australasian ecotoxicity database (AED) quality assessment scheme (Markich etal. 2002).	18	Yes^*c*^	None	No	Environmental toxicology research
Marshall etal. 2005	Derived from previously developed clinically based risk of bias assessment instruments or citing clinical studies supporting the inclusion of specific criteria; this instrument was based on CONSORT.	10	No	Shock/sepsis	No	Preclinical drug research
van der Worp etal. 2005 (Utrecht criteria)	Derived by modifying or updating previously developed animal research methodology assessment instruments or citing animal studies supporting the inclusion of specific criteria. The checklist was derived from the STAIR criteria (STAIR 1999), and recommendations resemble the scale used by Horn etal. (2001).	9	Yes	Stroke	No	Preclinical drug research
de Aguilar-Nascimento 2005	Derived by modifying or updating previously developed animal research methodology assessment instruments or citing animal studies supporting the inclusion of specific criteria; motivated by past research describing the importance of certain study design features (Festing 2003; Festing and Altman 2002; Johnson and Besselsen 2002).	9	No	None	No	General animal research
Macleod etal. 2004	Derived by modifying or updating previously developed animal research methodology assessment instruments or citing animal studies supporting the inclusion of specific criteria; informed by previously published criteria (Horn etal. 2001; Jonas etal. 1999).	10	Yes^*d*^	Stroke	Yes	Preclinical drug research
Bebarta etal. 2003	Derived from previously developed clinically based risk of bias assessment instruments or citing clinical studies supporting the inclusion of specific criteria; randomization and blinding were included based on evidence from human clinical trials showing that lack of these features often overestimates the magnitude of treatment effects.	2	No	None	Yes	Preclinical drug research
Verhagen etal. 2003	No description of how the instrument was developed.	10	No	None	No	General animal research
Festing and Altman 2002	Developed based on consensus and citing past guidelines; derived from published guidelines for contributors to medical journals (Altman etal. 2000), *invitro* models (Festing 2001), and a previously published checklist (Festing and van Zutphen 1997).	10	No	None	No	General animal research
Johnson and Besselsen 2002	No description of how the instrument was developed.	7	No	None	No	General animal research
Lucas etal. 2002	Derived by modifying or updating previously developed animal research methodology assessment instruments or citing animal studies supporting the inclusion of specific criteria. An 8-point rating system was developed based on two previous recommendations (Horn etal. 2001; STAIR 1999).	8	Yes^*d,e*^	None	Yes	Preclinical drug research
Horn etal. 2001 (Amsterdam criteria)	Derived by modifying or updating previously developed animal research methodology assessment instruments or citing animal studies supporting the inclusion of specific criteria; derived in part from the original STAIR guidelines (STAIR 1999).	8	Yes^*f*^	Stroke	No	Preclinical drug research
Durda and Preziosi 2000	Derived by modifying or updating previously developed animal research methodology assessment instruments or citing animal studies supporting the inclusion of specific criteria; compiled methodological requirements and acceptance criteria for ecotoxicology testing published by national and international governmental and testing organizations.	15	No	None	No	Environmental toxicology research
Klimisch etal. 1997	No description of how the instrument was developed.	9	No	None	No	Environmental toxicology research
Hsu 1993	No description of how the instrument was developed.	6	No	Stroke	No	Preclinical drug research
^***a***^Although no specific methodological score was proposed, the authors did rank their criteria based on their relative importance. The authors also favor a scoring system that could be used to assign credits/points each time a criterion is present in a study and proposed several ideas for how to assign scores. ^***b***^Development of the methodological scores was based on previous studies (Minnerup etal. 2008, 2009). To calculate a quality score, one point was awarded for each quality assessment criterion that was mentioned in a study. ^***c***^To calculate the quality score, points were awarded if the assessment criteria were satisfied in the article. The scores given for each question were added to give an overall score, which was expressed as a percentage of the total possible score. Data were classified as unacceptable (≤50%), acceptable (51–79%), or high (≥80%). ^***d***^To calculate the methodological score, one point was given for each criterion mentioned in the article. ^***e***^Studies containing total quality scores <5 were considered to be of “poor methodological quality”; studies with 5 or 6 points were considered to have “moderate methodological quality”; and studies with 7 or 8 points were considered to have “good methodological quality.” ^***f***^To calculate the methodological score, one point was given for each criterion mentioned in the article. Studies scoring <4 were considered to be of “poor methodological quality,” and studies scoring ≥4points were considered to be of “good methodological quality.”						

Six instruments contained at least one criterion that showed an association of the criterion with inflated drug efficacy in animal models.

Seven instruments calculated a score for assessing methodological “quality.” Descriptions of how these scores were calculated are provided in Table 1. Sixteen of the instruments were designed for no specific disease model; the most commonly modeled disease was stroke (9 of 30 instruments).

Only 1 instrument was tested for validity (Sena et al. 2007), and 1 instrument was tested for reliability (Hobbs et al. 2005). Overall, 18 instruments were designed specifically to evaluate preclinical drug studies, 8 instruments documented general animal research guidelines, and 4 instruments were designed to assess environmental toxicology research.

The total number of risk of bias, methodological, and/or reporting criteria assessed by each instrument ranged from 2 to 25. Table 2 shows the study design criteria used to assess risk of bias for each of the 30 instruments. Although these criteria were included in at least some of the instruments, they were not all supported by empirical evidence of bias. Blinding and randomization were the two most common criteria found in existing instruments; 25 instruments included randomization and 23 instruments included blinding. The need to provide a sample size calculation was listed in 18 instruments. None of the instruments contained all 13 criteria from our initial list; 2 instruments contained 9 criteria, and 4 instruments contained only 1 or 2 of the criteria.

**Table 2:** Study design criteria aimed at reducing bias by instrument.

Instrument reference	Random allocation of treatment	Allocation concealment	Blinding	Inclusion exclusion criteria stated	Sample size calculation	Compliance with animal welfare requirements	Conflict of interest disclosed	Statistical model explained	Animals with comorbidity	Test animal details	Dose–response model	Every animal accounted for	Optimal time window used	No. (%) of criteria in each instrument (*n*=13)
Vesterinen etal. 2011^*a*^	Y	Y	Y	Y	Y	N	Y	Y	N	Y	N	Y	N	9 (69)
Agerstrand etal. 2011^*a*^	Y	N	N	N	N	N	N	Y	N	Y	Y	N	Y	5 (38)
National Research Council Institute for Laboratory Animal Research 2011^*a*^	Y	N	Y	Y	N	N	N	N	N	Y	N	Y	N	5 (38)
Lamontagne etal. 2010^*a*^	Y	Y	Y	N	Y	N	N	N	Y	N	N	N	N	5 (38)
Conrad and Becker 2010^*a*^	N	N	N	N	N	N	Y	N	N	N	N	N	N	1 (8)
Vesterinen etal. 2010	Y	N	Y	N	Y	Y	Y	N	N	N	N	N	N	5 (38)
Kilkenny etal. 2010^*a*^	Y	N	Y	N	Y	Y	Y	Y	N	Y	N	N	N	7 (54)
Minnerup etal. 2010^*a*^	Y	N	Y	N	N	Y	Y	N	Y	Y	N	N	N	6 (46)
Hooijmans etal. 2010^*a*^	Y	N	Y	Y	Y	Y	N	Y	N	Y	N	Y	N	8 (62)
van der Worp etal. 2010^*a*^	Y	Y	Y	Y	Y	N	N	Y	N	N	N	Y	N	7 (54)
Macleod etal. 2009^*a*^	Y	Y	Y	Y	Y	N	Y	N	N	Y	N	Y	N	8 (62)
Fisher etal. 2009^*a*^	Y	Y	Y	Y	Y	N	Y	Y	Y	N	Y	N	N	9 (69)
Rice etal. 2008^*a*^	Y	N	Y	N	Y	N	N	N	N	Y	N	Y	N	5 (38)
Sniekers etal. 2008^*a*^	N	N	Y	N	Y	N	N	N	N	Y	N	N	Y	4 (31)
Sena etal. 2007^*a*^	Y	Y	Y	N	Y	Y	Y	N	Y	N	Y	N	N	8 (62)
Unger 2007	Y	N	Y	N	N	N	N	Y	N	N	N	Y	N	4 (31)
Hobbs etal. 2005^*a*^	N	N	N	N	N	N	N	Y	N	Y	Y	N	N	3 (23)
Marshall etal. 2005^*a*^	Y	N	Y	N	Y	N	N	N	N	Y	N	Y	N	5 (38)
van der Worp etal.2005^*a*^	Y	N	Y	N	Y	N	N	N	Y	N	N	N	N	4 (31)
de Aguilar- Nascimento 2005^*a*^	Y	N	Y	N	Y	N	N	N	N	N	N	N	N	3 (23)
Macleod etal. 2004^*a*^	Y	N	Y	N	Y	Y	Y	N	N	N	N	N	N	5 (38)
Bebarta etal. 2003	Y	N	Y	N	N	N	N	N	N	N	N	N	N	2 (15)
Verhagen etal. 2003^*a*^	N	N	N	N	N	N	N	Y	N	N	Y	N	N	2 (15)
Lucas etal. 2002^*a*^	Y	N	Y	N	N	N	N	N	N	N	Y	N	N	3 (23)
Festing and Altman 2002^*a*^	Y	N	Y	N	Y	N	N	Y	N	Y	N	N	N	5 (38)
Johnson and Besselsen 2002^*a*^	Y	N	N	N	Y	N	N	Y	N	N	N	N	Y	4 (31)
Horn etal. 2001^*a*^	Y	N	Y	N	N	N	N	N	N	N	Y	N	N	3 (23)
Durda and Preziosi 2000^*a*^	Y	N	N	N	N	N	N	Y	N	Y	Y	N	N	4 (31)
Klimisch etal. 1997^*a*^	N	N	N	N	N	N	N	N	N	Y	Y	N	N	2 (15)
Hsu 1993^*a*^	Y	N	Y	N	Y	N	N	N	N	N	Y	N	N	4 (31)
No. (%) of instruments containing criterion (*n*=30)	25 (83)	6 (20)	23 (77)	6 (20)	18 (60)	6 (20)	9 (30)	12 (40)	6 (20)	14 (47)	10 (33)	7 (23)	3 (10)
Abbreviations: Y, the criterion was present; N, the criterion was not present. ^***a***^The instrument contained additional criteria (see Supplemental Material, TableS1).														

Additional criteria assessed by each instrument are listed in Supplemental Material, Table S1. Some of these criteria related to reporting requirements for the abstract, introduction, methods, results, and conclusions, rather than risk of bias criteria. These reporting criteria were not included in the count for the number of risk of bias criteria assessed by an instrument. For example, Kilkenny et al. (2010) stated that the ARRIVE Guidelines is a 20-criteria instrument. However, we consider the ARRIVE Guidelines as a 13-criteria instrument because 7 of the original criteria pertain to reporting requirements. Fourteen instruments contained criteria to describe animal housing, husbandry, or physiological conditions. Inclusion of these criteria is empirically supported by studies showing that changes in housing conditions affect physiological and behavioral parameters in rodents (Duke et al. 2001; Gerdin et al. 2012). Among instruments that did not specify the need to use randomization, 4 of 5 instruments stated that a control group should be used.

## Discussion

In this systematic review we identified 30 instruments for assessing risk of bias and other methodological criteria of animal research. Identifying bias, the systematic error or deviation from the truth in actual results or inferences (Higgins and Green 2008), in animal research is important because animal studies are often the major or only evidence that forms the basis for regulatory or further research decisions. Our review highlights the variability in the development and content of instruments that are currently used to assess bias in animal research.

Most of the instruments were not tested for reliability or validity. One notable exception is the CAMARADES (Collaborative Approach to Meta-Analysis and Review of Animal Data from Experimental Studies) instrument developed by Sena et al. (2007); these authors combined criteria from four previous instruments and showed that the instrument appears to have validity. Similarly, Hobbs et al. (2005) tested the reliability of a modified version of the Australasian ecotoxicity database (AED) instrument and found an improvement in reliability compared with the original AED instrument. Furthermore, most of the instruments were not developed on the basis of empirical evidence showing an association between specific study design criteria and bias in research outcomes. Only six instruments included criteria that were supported by data showing an association between a particular methodological criterion and effect size in animal studies (Bebarta et al. 2003; Lucas et al. 2002; Macleod et al. 2004; Sena et al. 2007; Sniekers et al. 2008; Vesterinen et al. 2010). Most of the instruments contain criteria based on expert judgment, and others extrapolate from evidence of risk of bias in human studies. In addition, seven instruments calculated a “quality score”; however, these scores are not considered a valid measure of risk of bias, and this practice should be discontinued (Juni et al. 1999).

Types of bias that are known to influence the results of research include selection, performance, detection, and exclusion. These biases have been demonstrated in animal studies, and methodological criteria that can protect against the biases have been empirically tested.

Selection bias, which introduces systematic differences between baseline characteristics in treatment and control groups, can be minimized by randomization and concealment of allocation. Lack of randomization or concealment of allocation in animal studies biases research outcomes by altering effect sizes (Bebarta et al. 2003; Macleod et al. 2008; Sena et al. 2007; Vesterinen et al. 2010). Performance bias is the systematic difference between treatment and control groups with regard to care or exposure other than the intervention (Higgins and Green 2008). Detection bias refers to systematic differences between treatment and control groups with regard to how outcomes are assessed (Higgins and Green 2008). Blinding of investigators can protect against performance bias, and there is substantial evidence that lack of blinding in a variety of types of animal studies is associated with exaggerated effect sizes (Bebarta et al. 2003; Sena et al. 2007; Vesterinen et al. 2010). Blinding of outcome assessors is a primary way of reducing detection bias. There are many ways to achieve adequate blinding in animal studies, such as having coded data (blinding to treatment assignment) analyzed by a statistician who is independent of the rest of the research team. Exclusion bias refers to the systematic difference between treatment and control groups in the number of animals that were included in and completed the study. Accounting for all animals used in the study and using intention-to-treat analysis can reduce exclusion bias (Marshall et al. 2005).

Some criteria included in the animal research assessment instruments are not associated with bias. For example, a statement of compliance with animal welfare requirements is a reporting issue. Sample size calculations are often included as a criterion in animal research assessment instruments, but bias is not the same as imprecision. Whereas bias refers to systematic error, imprecision refers to random error, meaning that multiple replications of the same study will produce different effect estimates because of sampling variation (Higgins and Green 2008). Although larger and more precise studies may give a more accurate estimate of an effect, they are not necessarily less biased. Furthermore, sample size calculations can be greatly affected by the underlying assumptions made for the calculation (Bacchetti 2010). Although a sample size calculation is not a risk of bias criterion, it is an important characteristic to consider in evaluating an overall body of evidence.

Some of the criteria listed in the instruments are unique to animal studies. For example, in preclinical drug research, testing animals with comorbidities is necessary to identify whether or not candidate drugs retain efficacy in light of additional health complications and to more closely resemble the health status of humans. Empirical evidence supports the use of this criterion because studies that included healthy animals instead of animals with comorbidities overestimated the effect sizes of experimental stroke interventions by > 10% (Crossley et al. 2008). For environmental chemicals, use of comorbid animals could result in the opposite influence on effect size (i.e., to decrease it), and considering this as a criterion is consistent with recommendations to evaluate the influence of biological factors that may influence risk (National Research Council 2009). Timing of exposure also influences study outcome (Benatar 2007; van der Worp et al. 2010; Vesterinen et al. 2010), and some effects may be observed only for exposures that occur during certain developmental periods (National Research Council 2009). Sex, the nutritional status of experimental animals, and animal housing and husbandry conditions (Duke et al. 2001; Gerdin et al. 2012) could also affect the response to an intervention or environmental chemical exposure, but these criteria should be studied to determine if they introduce a systematic bias in results. These unique criteria have not been sufficiently included in the study instruments; even if these criteria do not produce systematic bias, they should be clearly described and reported in animal studies to aid interpretation of the findings (Marshall et al. 2005).

Although some risk of bias criteria have been investigated primarily in human studies, they warrant consideration for animal studies. Reviews of clinical studies have shown that study funding sources and financial ties of investigators (including university- or industry-affiliated investigators) are associated with favorable research outcomes for the sponsors (Lundh et al. 2011). In that study, favorable research outcomes were defined as either increased effect sizes for drug efficacy studies, or decreased effect sizes for studies of drug harm. Selective reporting of outcomes and failure to publish entire studies is considered an important source of bias in clinical studies; however, little is known about the extent of this bias in animal research (Hart et al. 2012; Rising et al. 2008).

Further research should consider potential interactions between criteria for assessing risk of bias. Existing instruments have tested the association of study design criteria on effect size using univariate models. Multiple regression models should be used to ascertain the relationship between a study design criterion and effect size when taking into account other criteria in the model. Covariance between methodological criteria should also be examined. For example, randomized studies may be less likely to omit blinding than nonrandomized studies (van der Worp et al. 2010). Knowing the relative importance of these criteria will provide additional support for inclusion of specific criteria in risk of bias assessment instruments.

Most of the instruments identified for our study exclude some criteria that appear to be important for assessing bias in animal studies (e.g., allocation concealment). It is important to recognize that some authors purposely exclude certain criteria from their instruments to reduce complexity and unnecessary detail. The most complex instrument had 25 criteria (Agerstrand et al. 2011). The detailed level of reporting needed to apply the gold standard publication checklist (GSPC), which has 17 criteria, was one of the main criticisms against it (Hooijmans et al. 2010).

Because many journals now allow online publication of supplemental data, risk of bias assessment should be less limited by a lack of space for reporting detailed methods. Reporting of clinical research has improved because risk of bias assessments for systematic reviews and other purposes have become more prevalent and standards for reporting have been implemented by journals (Turner et al. 2012). Recent calls for reporting criteria for animal studies (Landis et al. 2012; National Research Council Institute for Laboratory Animal Research 2011) recognize the need for improved reporting of animal research. As happened for clinical research, reporting of animal research is likely to improve if risk of bias assessments become more common.

Many of the instruments identified in our review were derived to evaluate preclinical animal drug research, which could limit their potential application in environmental health research. Although selection, detection, and performance biases are relevant for all animal research, some of the preclinical instruments contain criteria specific for assessing the quality of stroke research, such as the “avoidance of anesthetics with marked intrinsic neuroprotective properties” (Macleod et al. 2008; Sena et al. 2007). On the other hand, investigation of an optimal time window for outcome assessment (National Research Council 2009), the timing of the exposure (National Research Council 2009), and measurement of outcomes that are sensitive to the exposure at the appropriate time (Wood 2000) are particularly important for assessing animal studies of environmental exposures.

*Study limitations.* A limitation of our study is that we may not have identified all published assessment instruments for animal research. Our inclusion criteria allowed only articles published in English; therefore, we may have missed some instruments published in other languages. Furthermore, because we limited our search to articles indexed in Medline, articles indexed exclusively in Embase or some other database would have been missed. However, both our consultation with a librarian and the large pool of studies identified through the electronic search suggest that it was comprehensive.

## Conclusions

In this review we identified a wide variety of instruments developed to evaluate animal studies. The individual criteria included in animal risk of bias assessment instruments should be empirically tested to determine their influence on research outcomes. Furthermore, these instruments need to be tested for validity and reliability. Finally, existing instruments (many of which were developed using stroke models) need to be tested on other animal models to ensure their relevance and generalizability to other systems.

## Supplemental Material

(475 KB) PDF
