# Supplementary material for: Instruments for Assessing Risk of Bias and Other Methodological Criteria of Published Animal Studies: A Systematic Review
Source: Environ Health Perspect. 2013 Jun 14;121(9):985–92. doi: 10.1289/ehp.1206389 (PMC3764080; doi:10.1289/ehp.1206389)
Supplement: (475 KB) PDF [file ehp.1206389.s001.pdf]

Supplemental Material

Instruments for Assessing Risk of Bias and Other Methodological Criteria of Published Animal Studies: A Systematic Review

David Krauth, Tracey J. Woodruff, Lisa Bero

Corresponding author: Lisa Bero, PhD

Professor

Department of Clinical Pharmacy

Institute for Health Policy Studies University of California, San Francisco

Box 0613, 3333 California St, Suite 420

San Francisco, CA, USA 94118

Phone: (415) 476-1067

Fax: (415) 502-0792

berol@pharmacy.ucsf.edu

Table of Contents Page

Supplemental Material, Table S1. Additional Criteria found in Animal Research Assessment Instruments 2

References 6

Supplemental Material, Table S1. Additional Criteria found in Animal Research Assessment Instruments

**Instrument Identifier**	**Other Factors Measured by Assessment Instruments**	**Total Number of Additional Criteria**[a]
Vesterinen et al. 2011	Inclusion of primary research/hypothesis; Aim/purpose of the study should be clearly stated; Authors should also describe their study design and include a control group	3
Agerstrand et al. 2011	*Relevance Criteria* (n = 12 criteria): Use of a representative test substance; Use of relevant test substance for the risk assessment; Use of appropriate test species; Evaluation of appropriate life stages; Evaluation of appropriate endpoints; Use of relevant exposure route given the test species used; Test exposure scenario for tested substance? Relationship between the tested doses and environmental concentrations stated; Relevant and appropriate time of exposure for the endpoints studied; Description of environmental parameters that also influence the outcome (e.g. pH, temperature, light conditions); Accurate characterization of endpoint (adverse effect or not?); Reporting of all references.	20
	Reliability Criteria (n = 8 criteria): Purpose of study and endpoint described; Description of Protocol (e.g. standard, modified standard, etc.); Description of test compound; Description of dosing system (tested doses/concentrations, measured doses/concentrations, exposure duration, exposure route, exposure schedule, method for stock preparation, time point for observations); Description of control group; Description of test environment (pH, temperature, conductivity, etc.); Biological effects reported (i.e. results reproducible, results consistent with others); Other considerations (references to support study's reliability, produced according to GLP, raw data available).	
National Research Council (US) Institute for Laboratory Animal Research 2011	*Terrestrial animals* (n = 10 criteria): Use of a control group; Detailed description of food and feeding methods; Description of water source, deliver methods, and treatment (e.g. chlorination); Reporting of housing/husbandry conditions; Description of environmental parameters (lighting, temperature, humidity; relationship of dose administration to fasting); Description of anesthetics, analgesics, and other substances not part of the experimental treatment; Description of treatment administration (i.e. timing, frequency, route, buffering, and method); Description of infectious agents; Description of methods used to acquire tissue of body fluid samples; Description of method of euthanasia.	14
	Aquatic Systems (n = 4 criteria): Description of water quality parameters (i.e. temperature, ammonia, nitrite, nitrate, pH, dissolved oxygen, carbon dioxide, hardness, alkalinity, supersaturation, salinity, chlorine, chloramine, suspended solids, and heavy metals such as copper, zinc, and cadmium); Description of food (source, type, form, quantity, and nutrient and caloric content of diet); housing (includes type of system and lighting); Description of animal numbers (including stocking density, male to female sex ratio)	
Lamontagne et al. 2010	Completeness of follow-up; Intention-to-treat analysis; model of illness (e.g. infectious vs. non-infectious sepsis models, chronic versus acute sepsis models); Description of therapeutic intervention (timing, of intervention, administration of supportive measures)	4

Instrument Identifier	Other Factors Measured by Assessment Instruments	Total Number of Additional Criteria[a]
Conrad and Becker 2010	Principal investigator is legally guaranteed the following: freedom to publish, authority to analyze, interpret results, control study design; Public release of data and methods; Whether investigator adhered to accepted methods of scientific inquiry; Whether the study was included on a public registry of research for policy use; Whether the investigator's compensation was tied with a specific outcome; Whether the principal investigator agreed with the sponsor to give out his/her name for publication/presentation that was actually drafted by someone else; Whether or not investigators who work at multiple sites (e.g. academic institution and non-academic entity) maintain clarity about their affiliations when publishing reports; Whether or not sponsoring agency promotes use of systematic external review of research as a way to foster scientific integrity; Whether or not the article was peer-reviewed	9
Kilkenny et al. 2010	Study design description (number of experimental, control groups); Provide precise details of all procedures carried out; Housing and husbandry; Experimental outcomes (define clearly primary, secondary experimental outcomes assessed); Results should be generalizable to other animals or systems, including humans; Rationale for using specific animal model should be included. *The ARRIVE Guidelines also include criteria associated with reporting, including specific details for how to report the title, abstract, introduction, results, and discussion.*	6
Minnerup et al. 2010	Optimal (i.e. therapeutic) time window of treatment; Monitoring of physiological parameters; Assessment of two outcomes; Outcome assessment in acute phase; Outcome assessment in chronic phase.	5
Hooijmans et al. 2010	Rationale for using specific animal model should be stated; Type of experimental design stated; Study should contain both experimental and a control group; Adequate housing/husbandry; Nutrition requirements (food given to animals, including amount and time of day fed) should be documented; Water requirements (schedule, type, frequency of change) should be documented; Description of intervention should be provided; Inclusion of physiological parameters; Clear, specific/focused research question and hypothesis stated. *The Gold Standard Publication Checklist also includes criteria associated with reporting, including specific details for how to report the methods, results, and discussion.*	9
van der Worp et al. 2010	Monitoring of physiological parameters; Control of study conduct (to determine whether a third party controlled which conducts of the study)	2
Macleod et al. 2009	Study funding	1
Fisher et al. 2009	Therapeutic time window of treatment; Multiple outcomes measured (including histological and behavioral outcomes); Monitoring of physiological parameters; Treatment efficacy should be tested with two or more species; Results need to be replicated in at least one independent lab; Relevant biomarkers should be included	6
Rice et al. 2008	Housing/husbandry details	1
Sniekers et al. 2008	Appropriate controls (matched to experimental group); Treatment well described; Reliable outcome measurements (i.e. measurements should be validated, generally accepted).	3

Instrument Identifier	Other Factors Measured by Assessment Instruments	Total Number of Additional Criteria[a]
Sena et al. 2007	Monitoring of physiological parameters; Whether or not the article was peer-reviewed; Control of temperature; Avoidance of anaesthetics with intrinsic neuroprotective properties (specific to stroke therapy); Optimal (i.e. therapeutic) time window of treatment; Functional outcome assessment; Histological outcome assessment; Results replicated in 2 labs; Tested in models of permanent and temporary occlusion; Tested in males and females; Use of clinically appropriate administration route; Assessment in acute phase; Assessment in chronic phase	13
Hobbs et al. 2005	Duration of exposure stated; Type (static, flow through) of exposure stated; Biological endpoint stated and defined; Biological effect stated; Biological effect quantified; Use of appropriate controls; Duplication of control and chemical concentration; Was the test acceptability criteria stated or inferred?; Description of test media; Measurement of chemical concentrations conducted; Parallel reference toxicant toxicity tests conducted; Water quality parameters measured (pH, hardness; alkalinity, organic carbon concentration); Description of salinity/conductivity conditions stated for marine and estuarine water; Description of dissolved oxygen of the test water stated for tests not using aquatic macrophytes and alga; Temperature measured and stated; Use of highest possible purity chemical or analytical reagent grade chemicals for the experiment	15
Marshall et al. 2005	Use of control group; Animal housing/husbandry; Multiple (i.e. primary/secondary) endpoints; Intention-to-treat analysis should be performed; Co interventions should be documented	5
van der Worp et al. 2005	"Clinically relevant time window for start of treatment" (i.e. treatment administered 60 minutes after ishaemia onset) (Simon and Shiraishi 1990); Monitoring of physiological parameters; Assessment of multiple outcomes; Outcome assessment in acute phase; Outcome assessment in chronic phase	5
Macleod et al. 2004	Statement of Control of Temperature; Avoidance of anesthetics with intrinsic neuroprotective properties (specific to stroke therapy); Blinded induction of ischemia (specific to stroke therapy); Appropriate animal model (aged, diabetic, or hypertensive) should be used ; Whether or not the article was peer-reviewed	5
Verhagen et al. 2003	Hypothesis Driven (a plausible hypothesis and supportive mechanism); Valid test system should be used, including the use of an appropriate control); Route of administration coincides with human exposure pathways; GLP not required but an advantage; Test substance should be standardized using analytical techniques or by biological effects; Investigators should use known dose level (substantiated from data from past studies) that induces the desired toxic effect; Multiple variables are generally necessary for in vivo systems (histological and clinical variables); Repeatability/Reproducibility (repeatability not required for in-vivo studies)	8
Lucas et al. 2002	Optimal (i.e. therapeutic) time window of treatment; Monitoring of physiological parameters; Multiple outcomes assessed; Outcome assessment in acute phase; Outcome assessment in chronic phase	5
Festing and Altman 2002	Use of a control group; Clearly stated research objectives/hypothesis; Rationale for choosing specific animal model should be provided; housing/husbandry details; nutrition/diet requirements should be documented	

Instrument Identifier	Other Factors Measured by Assessment Instruments	Total Number of Additional Criteria[a]
Johnson and Besselsen 2002	Use of a control group; Clear objectives/hypothesis stated; Rationale for specific animal model should be stated	3
Horn et al. 2001	Optimal (i.e. therapeutic) time window of treatment investigated; Monitoring of physiological parameters; Assessment of at least two outcomes; Outcome assessment in acute phase; Outcome assessment in chronic phase	5
Durda and Preziosi 2000	Hypothesis clearly described; Appropriate endpoints for hypothesis; Description of protocol used; Description of test compound (chemical source, chemical species, purity/stability, vehicle); Description of dosing system (dose [measured preferred], administration route [environmentally relevant preferred], exposure schedule, exposure duration); Description of controls (i.e. control media identical to test media except for treatment, control and test organism from same population, use of acceptable control mortality/morbidity, vehicle control, positive/negative control); Description of test environment (lighting, water characteristics [pH, dissolved oxygen, etc.], physical structure of test environment described), feeding/food requirements); Quantitative measurement of response (preferred); Peer-reviewed (preferred); Results reproduced by others (preferred); Consistent with other findings (preferred)	11
Klimisch et al. 1997	Sample size included (Does not say calculation is needed though); Control group included; Purity/composition/origin of the test substance; Scope of the investigations per animal; Description of route/doses of administration; Description of test condition; Description of changes/lesions observed	7
Hsu 1993	Use of a placebo; Overall assessment of the outcome should include morbidity and mortality	2

[a] The total number of additional criteria does not include criteria associated with reporting, such as the reporting criteria contained in the ARRIVE Guidelines.

References

Agerstrand M, Kuster A, Bachmann J, Breitholtz M, Ebert I, Rechenberg B, et al. 2011. Reporting and evaluation criteria as means towards a transparent use of ecotoxicity data for environmental risk assessment of pharmaceuticals. Environ Pollut 159(10):2487-2492.

Conrad JW, Jr., Becker RA. 2010. Enhancing credibility of chemical safety studies: emerging consensus on key assessment criteria. Environ Health Perspect 119(6):757-764.

Durda JL, Preziosi DV. 2000. Data Quality Evaluation of Toxicological Studies Used to Derive Ecotoxicological Benchmarks. Human and Ecological Risk Assessment: An International Journal 6(5):747-765.

Festing MF, Altman DG. 2002. Guidelines for the design and statistical analysis of experiments using laboratory animals. ILAR J 43(4):244-258.

Fisher M, Feuerstein G, Howells DW, Hurn PD, Kent TA, Savitz SI, et al. 2009. Update of the stroke therapy academic industry roundtable preclinical recommendations. Stroke 40(6):2244-2250.

Hobbs DA, Warne MSJ, Markich SJ. 2005. Evaluation of Criteria Used to Assess the Quality of Aquatic Toxicity Data. Integrated Environmental Assessment and Management 1(3):174 - 180.

Hooijmans CR, Leenaars M, Ritskes-Hoitinga M. 2010. A gold standard publication checklist to improve the quality of animal studies, to fully integrate the Three Rs, and to make systematic reviews more feasible. Altern Lab Anim 38(2):167-182.

Horn J, de Haan RJ, Vermeulen M, Luiten PG, Limburg M. 2001. Nimodipine in animal model experiments of focal cerebral ischemia: a systematic review. Stroke 32(10):2433-2438.

Hsu CY. 1993. Criteria for valid preclinical trials using animal stroke models. Stroke 24(5):633-636.

Johnson PD, Besselsen DG. 2002. Practical aspects of experimental design in animal research. ILAR J 43(4):202-206.

Kilkenny C, Browne WJ, Cuthill IC, Emerson M, Altman DG. 2010. Improving bioscience research reporting: the ARRIVE guidelines for reporting animal research. PLoS Biol 8(6):e1000412.

Klimisch HJ, Andreae M, Tillmann U. 1997. A systematic approach for evaluating the quality of experimental toxicological and ecotoxicological data. Regul Toxicol Pharmacol 25(1):1-5.

Lamontagne F, Briel M, Duffett M, Fox-Robichaud A, Cook DJ, Guyatt G, et al. 2010. Systematic review of reviews including animal studies addressing therapeutic interventions for sepsis. Crit Care Med 38(12):2401-2408.

Lucas C, Criens-Poublon LJ, Cockrell CT, de Haan RJ. 2002. Wound healing in cell studies and animal model experiments by Low Level Laser Therapy; were clinical studies justified? a systematic review. Lasers Med Sci 17(2):110-134.

Macleod MR, O'Collins T, Howells DW, Donnan GA. 2004. Pooling of animal experimental data reveals influence of study design and publication bias. Stroke 35(5):1203-1208.

Macleod MR, Fisher M, O'Collins V, Sena ES, Dirnagl U, Bath PM, et al. 2009. Good laboratory practice: preventing introduction of bias at the bench. Stroke 40(3):e50-52.

Marshall JC, Deitch E, Moldawer LL, Opal S, Redl H, van der Poll T. 2005. Preclinical models of shock and sepsis: what can they tell us? Shock 24 Suppl 1:1-6.

Minnerup J, Wersching H, Diederich K, Schilling M, Ringelstein EB, Wellmann J, et al. 2010. Methodological quality of preclinical stroke studies is not required for publication in high-impact journals. J Cereb Blood Flow Metab 30(9):1619-1624.

National Research Council (US) Institute for Laboratory Animal Research. 2011. Guidance for the Description of Animal Research in Scientific Publications 2012/03/02 ed. Washington D.C.: The National Academies Press.

Rice AS, Cimino-Brown D, Eisenach JC, Kontinen VK, Lacroix-Fralish ML, Machin I, et al. 2008. Animal models and the prediction of efficacy in clinical trials of analgesic drugs: a critical appraisal and call for uniform reporting standards. Pain 139(2):243-247.

Sena E, van der Worp HB, Howells D, Macleod M. 2007. How can we improve the pre-clinical development of drugs for stroke? Trends Neurosci 30(9):433-439.

Simon R, Shiraishi K. 1990. N-methyl-D-aspartate antagonist reduces stroke size and regional glucose metabolism. Ann Neuorl 27(6):606-611.

Sniekers YH, Weinans H, Bierma-Zeinstra SM, van Leeuwen JP, van Osch GJ. 2008. Animal models for osteoarthritis: the effect of ovariectomy and estrogen treatment - a systematic approach. Osteoarthritis Cartilage 16(5):533-541.

van der Worp HB, de Haan P, Morrema E, Kalkman CJ. 2005. Methodological quality of animal studies on neuroprotection in focal cerebral ischaemia. J Neurol 252(9):1108-1114.

van der Worp HB, Howells DW, Sena ES, Porritt MJ, Rewell S, O'Collins V, et al. 2010. Can animal models of disease reliably inform human studies? PLoS Med 7(3):e1000245.

Verhagen H, Aruoma OI, van Delft JH, Dragsted LO, Ferguson LR, Knasmuller S, et al. 2003. The 10 basic requirements for a scientific paper reporting antioxidant, antimutagenic or anticarcinogenic potential of test substances in in vitro experiments and animal studies in vivo. Food Chem Toxicol 41(5):603-610.

Vesterinen HM, Egan K, Deister A, Schlattmann P, Macleod MR, Dirnagl U. 2011. Systematic survey of the design, statistical analysis, and reporting of studies published in the 2008 volume of the Journal of Cerebral Blood Flow and Metabolism. J Cereb Blood Flow Metab 31(4):1064-1072.
